# Direct femoral head approach without surgical dislocation for femoral head chondroblastoma: a report of two cases

**DOI:** 10.1186/s12893-022-01766-x

**Published:** 2022-08-29

**Authors:** Hirohisa Katagiri, Mitsuru Takahashi, Hideki Murata, Junji Wasa, Michihito Miyagi, Yosuke Honda

**Affiliations:** Division of Orthopaedic Oncology, Shizuoka Cancer Center, 1007 Shimonagakubo, Nagaizumi-cho, Sunto-gun, Shizuoka Prefecture 411-8777 Japan

**Keywords:** Chondroblastoma, Femoral head, Surgical dislocation, Long-term result, Surgery

## Abstract

**Background:**

Chondroblastomas are rare, benign, locally aggressive lesions that appear in the epiphysis. Surgery for femoral head chondroblastoma (FHCB) is difficult. Conventional treatment with curettage via a drilled tunnel along the femoral neck can damage the growth plate and is associated with high local recurrence rates. The trapdoor procedure, which directly facilitates lesion access from the femoral head articular surface, can reduce local recurrence and avoid growth plate damage, although it requires surgical dislocation. Little is known about the long-term results of this direct articular surface approach, and there are no case reports on trapdoor procedures without dislocation.

**Case presentation:**

We report two cases (patients aged 12 and 15 years) of FHCB presented with coxalgia treated using the trapdoor procedure without surgical dislocation. Both surgeries were performed with patients in the semi-lateral position. The hip joint was exposed via an anterior approach, and a capsulotomy was performed at the superior rim of the acetabulum, followed by the external rotation of the hip joint. With a fine osteotome, a rectangular flap (trapdoor) was opened on the cartilage surface in the lateral non-weight-bearing area, and curettage of the lesion followed by bone and/or bone substitute grafting was performed. Subsequently, the trapdoor was replaced in its original position. There has been no local recurrence or femoral head aseptic necrosis after more than 6 and 12 years for patients 1 and 2, respectively. Both patients had musculoskeletal tumor society scores of 100% at follow-up and are enjoying a normal active life.

**Conclusions:**

This direct femoral head approach without dislocation may be a simple treatment alternative for FHCB.

## Background

Chondroblastomas are rare, benign, bone tumors that represent approximately 1–2% of all primary bone tumors and 4–9% of all benign bone tumors. Chondroblastomas usually arise at the epiphysis, and the majority of cases occur in the second decade of life [1–3]. The most common sites are the proximal humerus and the proximal tibia, followed by the distal femur, and proximal femur [1]. In the proximal femur, chondroblastomas involving the greater trochanteric apophysis occur more often and are easily accessible to curettage [2].

However, chondroblastomas involving the epiphysis of the femoral head present a therapeutic dilemma regarding the optimal means of access, because the proximal femoral epiphysis is completely intracapsular and access to the lesion would involve a transgression of either the growth plate or the articular cartilage. The surgical choices, therefore, have been recommended as follows: (1) curettage via a tunnel drilled along the femoral neck (CVFN) with potential damage to the epiphyseal growth plate and high local recurrence rate [2–4], (2) an open approach with capsulotomy and making a cortical window for curettage at the femoral neck just below the femoral head [4], or (3) a trapdoor procedure, which is a direct approach through the articular surface of the femoral head after hip joint dislocation, which was first described by Iwai et al. in 2008 [5].

Following the first case report of the trapdoor procedure, modified techniques that included accessing the lesions from the attachment of the ligamentum teres on the fovea of the femoral head, instead of from the articular surface, were reported [6, 7]. The trapdoor procedure can avoid growth plate damage but can potentially damage the articular cartilage and is associated with a risk of femoral head avascular necrosis (AVN) from surgical dislocation. Long-term results of the direct approach from the articular surface have not been reported except for the original trapdoor procedure reported in one case report and again discussed in a series by Strong et al. where the surgery was done for a recurrent lesion [4, 5]. The former patient underwent curettage following surgical dislocation; however, details on the latter patient were not described in the study. Both patients experienced excellent results. To lessen the periarticular damage and complications from dislocation, we treated two patients by the direct femoral head approach without surgical dislocation (trapdoor procedure without dislocation) for curettage of femoral head chondroblastoma (FHCB). The purpose of this study is to describe our modified technique and its long-term results.

## Case presentation

### Case 1

**Fig. 1:**
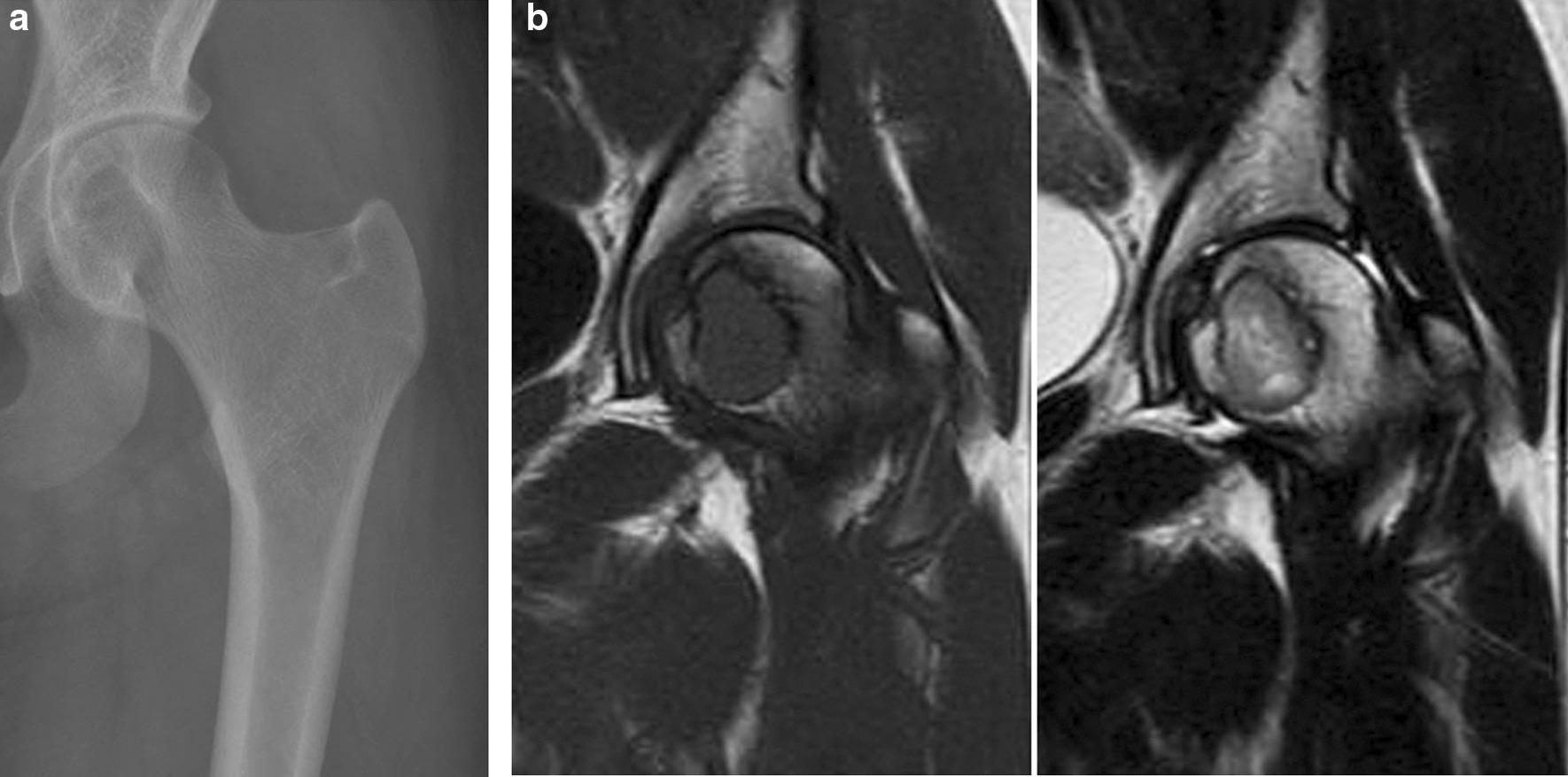
Preoperative images: Case 1. **a** X-ray image showing a 2 cm × 2 cm osteolytic lesion with a marginal sclerotic rim. **b** MRI revealing a lesion that is low intensity on T1 (left) and heterogeneously high intensity on T2 (right) imaging, with perilesional edema

A 15-year-old girl was referred to our hospital with a history of atraumatic left coxalgia, which had been present for 5 months. She had no family or medical history of any particular note. Physical examination revealed a decreased range of motion (ROM) at the hip on external rotation and abduction. Plain X-ray images revealed a 2 cm × 2 cm osteolytic lesion in the femoral head with a marginal sclerotic rim (Fig. 1a). Magnetic resonance imaging (MRI) showed a lesion of low signal intensity on a T1 weighted image and high signal intensity on a T2 weighted image with perilesional bone edema (Fig. 1b). The lesion was well enhanced by gadolinium contrast. Computed tomography (CT) scanning revealed a lesion located just below the subchondral bone that encroached toward the metaphysis of the femoral neck and displayed lobular growth and marginal sclerosis. An open biopsy of the femoral head was performed via the femoral neck. On histopathology, the tumor consisted predominantly of well-defined round or polygonal chondroblast-type cells. There were multi-nucleated osteoclast-type giant cells, foci of the chondroid matrix, and a fine network of pericellular “chicken wire” calcifications; therefore, the patient was diagnosed with a chondroblastoma.

### Surgical technique

**Fig. 2:**
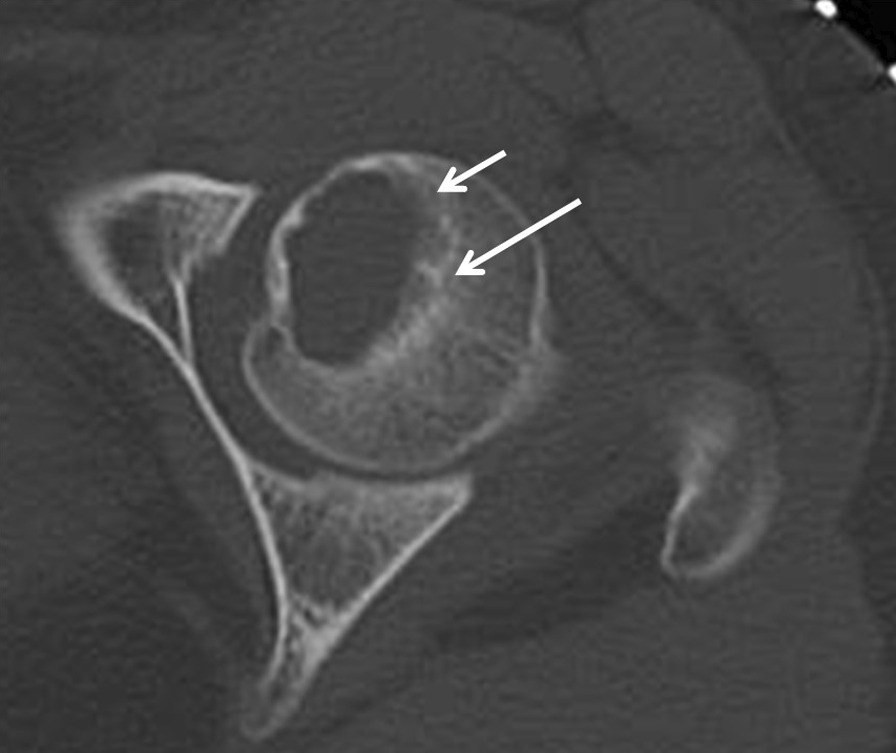
Preoperative computed tomography (CT) imaging: Case 1. CT images revealing a lytic lesion with a sclerotic rim located in the anteromedial side of the femoral head. The white arrow indicates the direction in which the osteotome was driven

**Fig. 3:**
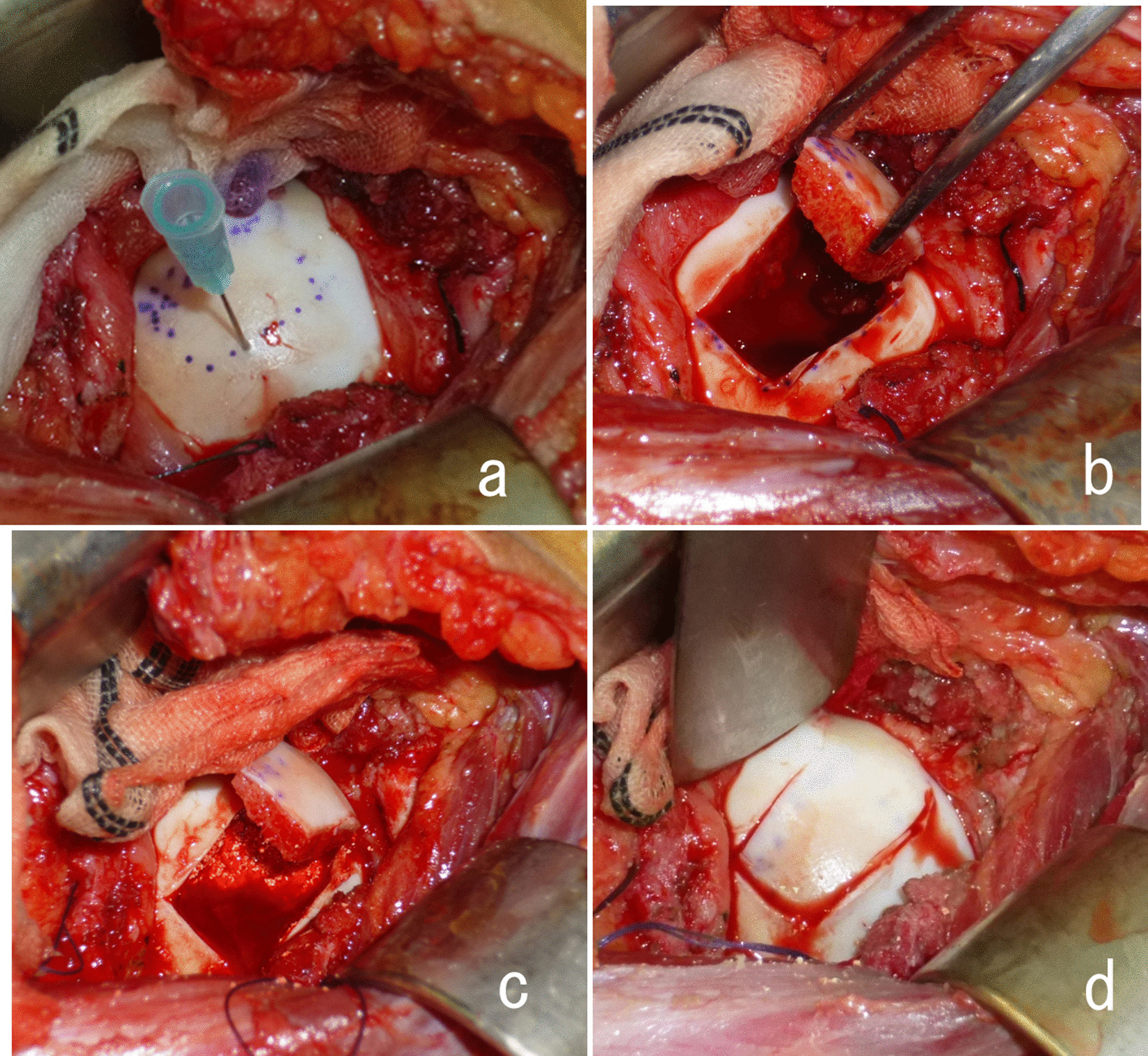
Images obtained during surgery in Case 1. **a** Exposed articular surface of the femoral head without dislocation. **b** The trapdoor segment was lifted, and thorough excision curettage was performed. **c** Autologous cancellous bone and bone substitute was grafted. **d** The door was closed

**Fig. 4:**
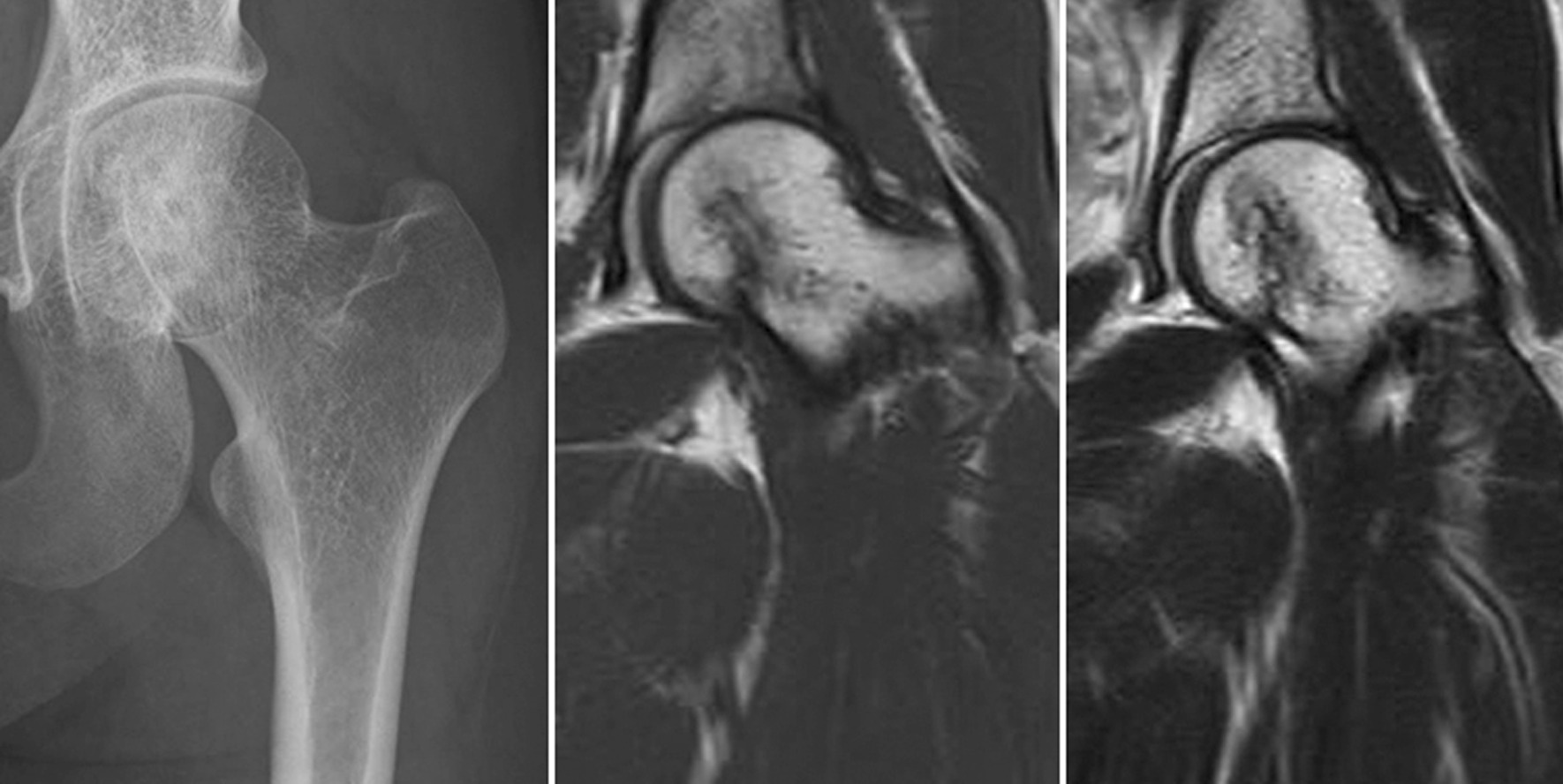
Imaging findings obtained 5 years postoperatively: Case 1. Plain X-ray image (left), T1- and T2-weighted (middle and right, respectively) MRI images demonstrating that there is no recurrence or avascular necrosis

The patient was placed in a semi-lateral position with the affected side elevated by a cushion placed underneath the buttock. The hip joint was exposed via an anterior approach between the sartorius and tensor fasciae lata. A T-shaped capsulotomy was performed at the superior rim of the acetabulum. The hip joint was externally rotated maximally without dislocation. The location of the subchondral lesion was detected using a preoperative CT scan that was obtained with the limb in the maximum externally rotated position, which was intraoperatively replicated (Fig. 2). With a fine osteotome, a rectangular flap of 1 cm × 1.8 cm (referred to hereafter as a trapdoor) was opened on the surface of the cartilage at the lateral non-weight-bearing area, followed by subchondral and cancellous bone dissection to access the lesion in the femoral head (Fig. 3a, b). The rectangular trapdoor was opened further to access the lesion site, and aggressive curettage was carried out; after that, autologous cancellous bone was harvested from the ipsilateral iliac crest and was used with a bone substitute (β-tricalcium phosphate [β-TCP]) for filling the osseous defect (Fig. 3c). Subsequently, the trapdoor was replaced (Fig. 3d), and the capsule was sutured with a fine absorbable thread. Postoperatively, ROM exercises and 5-kg weight-bearing were started after a few days. Weight-bearing on the affected lower limb was gradually increased, although full weight-bearing was not permitted until 2 months after surgery. There was no local recurrence or AVN at the postoperative follow-up after 6 years (Fig. 4). On follow-up, the patient did not have discomfort, pain, or ROM restriction and had a musculoskeletal tumor society (MSTS) score of 100% [8].

### Case 2

**Fig. 5:**
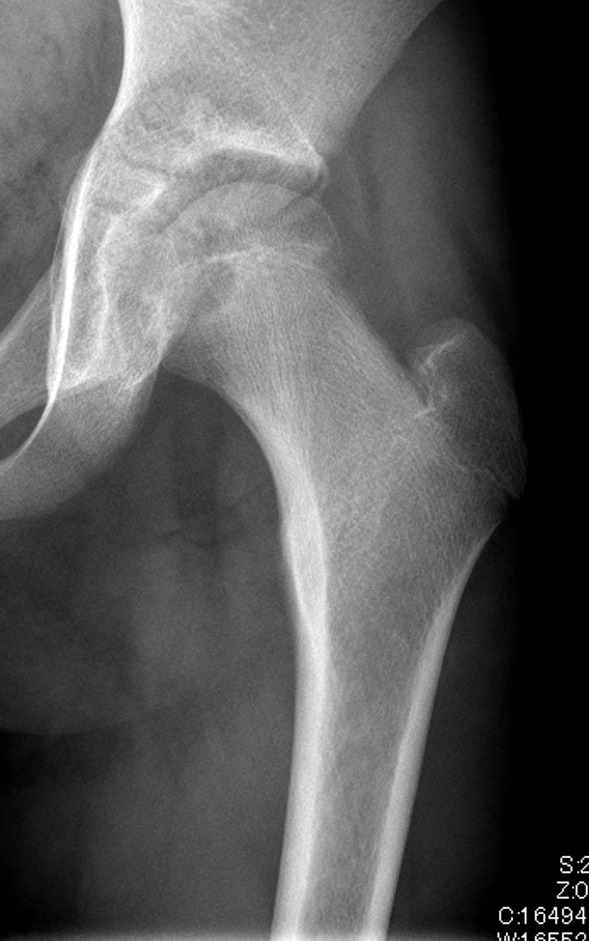
Preoperative X-ray images: Case 2. X-ray image showing a 1 cm × 1.5 cm osteolytic lesion with a marginal sclerotic rim

**Fig. 6:**
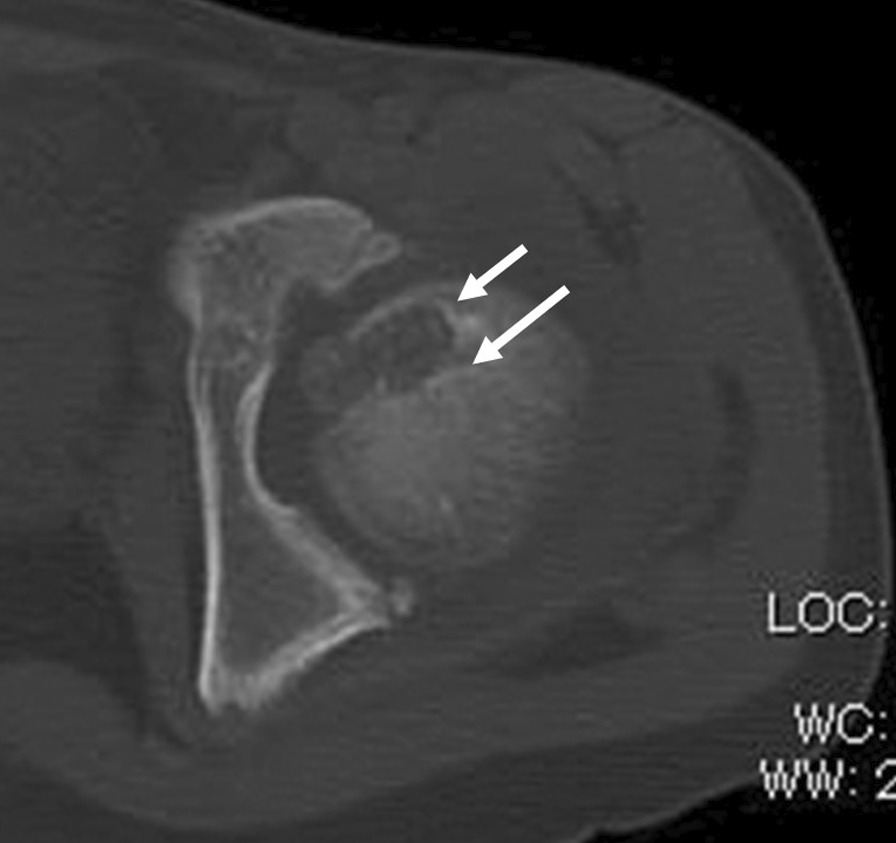
Preoperative computed tomography (CT) image: Case 2. CT images revealing a lytic lesion with a sclerotic rim located in the anteromedial side of the femoral head. The white arrow indicates the direction in which the osteotome was driven

**Fig. 7:**
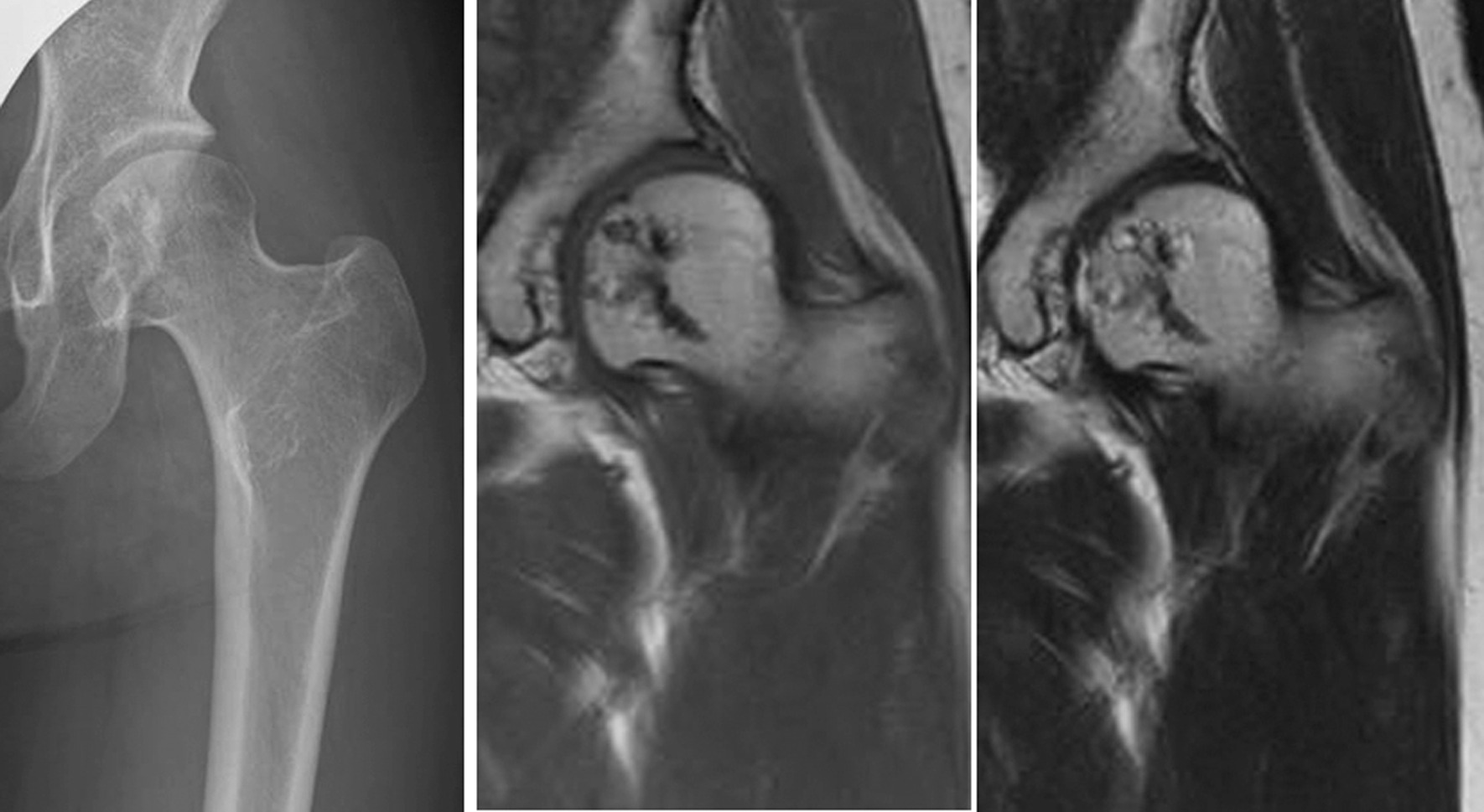
Imaging findings obtained 10 years postoperatively: Case 2. Plain X-ray image (left) and T1- and T2-weighted (middle and right, respectively) MRI images demonstrating that there is no recurrence or avascular necrosis

A 12-year-old girl presented to our hospital with a history of coxalgia for 4 months. She had no particular family or past history. Plain X-ray images revealed a 1 cm × 1.5 cm osteolytic lesion in the epiphysis of the femoral head and a physis that was still open (Fig. 5). An MRI of the lesion revealed it was well enhanced on gadolinium contrast and had associated peritumoral edema and excessive joint effusion. CT images revealed marginal sclerosis of the lesion (Fig. 6). A needle biopsy via the femoral neck was performed cautiously to minimize damage to the patient’s femoral growth plate; histopathologic examination revealed that the lesion was an FHCB. The surgical approach used for Case 1 was also employed for this patient. As the femoral head was small, a rectangular trapdoor had to be removed to facilitate curettage. After β-TCP grafting, the trapdoor was placed back in the original position. The postoperative rehabilitation was uneventful, and full weight-bearing was possible 2 months after the surgery. Twelve years after the surgery, there was no sign of recurrence or AVN (Fig. 7). On follow-up examination, the patient was asymptomatic, and her MSTS score was 100%.

## Discussion and conclusion

Chondroblastomas are benign but locally aggressive tumors that are usually treated with curettage of the lesion and reconstruction with allograft, autologous bone graft, or bone substitute packing. Local recurrence rates between 8 and 15% have been reported, suggesting that the tumor location can influence local recurrence [1–4, 9]. The clinical utility of adjuvant treatments, such as cryotherapy or phenols, is controversial because of the potential for damage to the joint cartilage, especially within the femoral head. Chondroblastomas usually occur in the epiphysis, but lesions within the femoral head pose a therapeutic challenge because the epiphysis of the femoral head is completely intracapsular, and approaching the femoral head confers an inherent risk for AVN and iatrogenic femoral head deformity, which likely leads to osteoarthritic changes of the hip joint in adolescence.

There have been four approaches to the management of FHCB. The first is conventional CVFN, which involves curettage through a tunnel drilled from the lateral trochanteric area. However, the tunnel needs to be sufficiently long to reach the epiphysis of the femoral head. Consequently, direct visualization of the tumor is difficult and usually requires C-arm control or arthroscopy via a bone tunnel. Thus, the curettage tends to be insufficient for CVFN and is associated with local recurrence rates as high as 40–50% [2–4]. The second procedure involves a direct approach for curettage through the femoral neck or head-neck junction below the growth plate following a capsulotomy, with an associated low recurrence rate. This procedure is similar to the one performed for an early stage of AVN. However, complete curettage may prove difficult with this method, even from the femoral head-neck junction, especially if the lesion extends to the anterior part of the femoral head. This is because the proximal femur is anatomically shaped to expand abruptly in a trumpet shape from the neck to the epiphysis, making it difficult to perform a complete curettage on the lesion even with a special curvature curette. Even if it can be done, it is likely to be insufficient as the condition cannot be observed under direct vision.

Further, although Strong et al. documented no AVN among five cases, this procedure may increase the risk for AVN because the main blood supply to the femoral head traverses this area [4, 10]. In addition, both methods mentioned above can damage the growth plate. The third procedure is radiofrequency ablation (RFA), which is a less invasive approach that has a low local recurrence rate (i.e., n = 1/17 patients, 6% [11]; n = 0/8 patients, 0% [12]; and n = 3/25 patients, 12% [13]). However, RFA can induce iatrogenic damage to the articular cartilage due to the high temperatures of the operation. Lalam et al. reported that articular damage was present in 2 out of 8 cases [12]. The follow-up period was too short in the study conducted by Xie et al. [13], and it was unclear how many lesions of the femoral head were included in their study [11, 13]. Petsas et al. reported two adult FHCB cases successfully treated with RFA following diagnostic curettage via a drilled tunnel in the femoral neck. However, the follow-up period was only 1 year and showed insufficient bone regeneration in one case [14]. These results suggest that the efficacy of RFA for FHCB remains inconclusive.

To adequately visualize the tumor for curettage and avoid growth plate damage, the fourth method, a direct approach from the articular surface represented by the trapdoor procedure, was first reported by Iwai et al. in 2008 [5]. This trapdoor technique was originally developed for the treatment of AVN and applied to treat FHCB [15]. This technique is a procedure for curettage through a “trapdoor” opened on the articular surface following a trochanteric osteotomy and surgical hip dislocation. The researchers reported that a subchondral lesion was confirmed visually as a depressed cartilaginous area without fluoroscopic guidance, and a rectangular door was created on it. Following that report, Xu et al. reported 13 patients and Liu et al. reported 17 patients successfully treated with a modified trapdoor procedure wherein the access to the lesion was opened around the ligamentum teres rather than on the articular surface, and closed by using this ligament [6, 7]. No cases of local recurrence occurred in their series; however, two cases of AVN and arthritis were reported. Farfalli et al. reported that joint degeneration occurred in five of eight FHCB cases treated with curettage [16]. Their surgical approach was to dislocate the hip carefully and raise a window either through the femoral neck, as described by Strong et al., or through the femoral head. These results and those of other studies indicate that even when performed carefully, the surgical dislocation of the hip and open approach from the femoral neck or head risk damaging the articular surface of the femoral head and increase the risk of AVN [16–18].

Our method of opening the trap door on the articular cartilage without dislocation is simple; however, we could not find any English literature clearly describing this procedure and little is reported regarding long-term results of direct articular surface approach. Laitinen et al. briefly mentioned about two cases treated by making a trapdoor on the articular surface in their series, but provided no information regarding the location of the trapdoor, necessity of dislocation, and follow-up period [2]. In our literature search, we found only two proper reports on the long-term results of trapdoor creation on the femoral articular surface, which is associated with a concern of osteoarthritic changes during adolescence. The first case was reported by Iwai et al., and the second one was included in a series of 10 cases reported by Strong et al., wherein a posterior approach was adopted, but a requirement for surgical dislocation was not reported. Both patients fully recovered after 5 and 9 years, respectively [4, 5]. Compared to Iwai and collaborators’ [5] original technique or modified approach via the ligamentum teres, our procedure approached the lesion from the lateral non-weight-bearing area instead of the area where the lesion was closest to the articular surface. This is where the articular surface is outside of the acetabulum and, therefore, accessible without surgical dislocation. Approaching the lesion from this lateral area ensures that cancellous bone is retained between the tumor and articular cartilage. A thin, sharp, and narrow osteotome, should be inserted from the anterolateral to the posteromedial direction, cutting the articular cartilage together with the subchondral and cancellous bone to reach the lesion (Figs. 2 and 6). The subchondral bone and cancellous bone that adhered to the lid of the articular cartilage enabled stable fitting after the closure of the “trap door.” Abducting the hip joint and maximally rotating the joint externally not only made the lesion more accessible from the anterolateral articular surface without dislocation, but also positioned the trapdoor on the inferior aspect of the anterolateral articular surface, which is a non-weight-bearing area.

However, our method has three limitations. First, it is not clear whether this approach is feasible in posteriorly located FHCBs since both the cases described in this report were of anteriorly located FHCB. Conventional curettage from the anterior aspect of the greater trochanter just lateral to the intertrochanteric line can be an alternative solution in cases of posteriorly located FHCBs if the epiphyseal plates have closed. Second, it is difficult to locate an optimal site for opening the trapdoor on the articular surface because this technique must be performed on normal-looking cartilage in the lateral non-weight-bearing area. To solve this problem, it is necessary to obtain preoperative CT images in the same alignment as the surgical position or to use an intraoperative navigation system. Third, FHCB is a rare condition, and this is a retrospective report of only two cases. Prospective, multicenter studies with larger study populations treated using this technique are needed to ascertain the clinical utility of this surgical approach conclusively.

In conclusion, we treated two patients with FHCB via a direct femoral head approach from the inferior aspect of the anterolateral articular surface, which is non-weight-bearing area, without surgical dislocation (trapdoor procedure without dislocation). There has been no reported local recurrence, AVN, or postoperative functional loss in our patients after a mean follow-up of 108 months. This direct femoral head approach without surgical dislocation may be considered as a simple solution to treat FHCB.

## Data Availability

The datasets during and/or analyzed during the current study are available from the corresponding author on reasonable request.

## Abbreviations

CVFN: Curettage via a tunnel drilled along the femoral neck
FHCB: Femoral head chondroblastoma
ROM: Range of motion
MRI: Magnetic resonance imaging
CT: Computed tomography
MSTS: Musculoskeletal tumor society
AVN: Femoral head avascular necrosis
RFA: Radiofrequency ablation
